# Development of a Genetically Engineered Porcine Model of Rhabdoid Tumor Predisposition Syndrome Type 1 (RTPS-1)

**DOI:** 10.3390/cancers18121879

**Published:** 2026-06-09

**Authors:** Brian Na, C. Dustin Rubinstein, Jennifer J. Meudt, Fausto J. Rodriguez, Brent P. Lehman, Jamie L. Reichert, Jeremie Vitte, Dhanansayan Shanmuganayagam, Marco Giovannini

**Affiliations:** 1 Department of Head and Neck Surgery, UCLA David Geffen School of Medicine, Los Angeles, CA 90095, USA; 2 Division of Neuro-Oncology, Department of Neurological Surgery, UCSF, San Francisco, CA 94143, USA; 3 Jonsson Comprehensive Cancer Center, UCLA David Geffen School of Medicine, Los Angeles, CA 90095, USA; fjrodriguez@mednet.ucla.edu; 4 Biotechnology Center, University of Wisconsin-Madison, Madison, WI 53706, USA; 5 Biomedical and Genomic Research Group, Department of Animal and Dairy Sciences, University of Wisconsin-Madison, Madison, WI 53706, USA; 6 Division of Neuropathology, Department of Pathology, UCLA David Geffen School of Medicine, Los Angeles, CA 90095, USA; 7 Swine Research and Teaching Center, Department of Animal and Dairy Sciences, University of Wisconsin-Madison, Madison, WI 53706, USA; 8 Department of Surgery, University of Wisconsin-Madison, Madison, WI 53706, USA

**Keywords:** pig model, CRISPR/Cas9, rhabdoid tumors

## Abstract

**Background and Objectives:** Among CNS malignancies arising in infancy, ATRT stands out as the most frequently diagnosed in children younger than six months. Disruption of the SMARCB1 gene underlies the overwhelming majority of cases. Progress toward effective treatment has been hampered by two persistent challenges. Current mouse models, while informative, fall short of reproducing the full clinical and biological picture of human ATRT, and their ability to predict therapeutic outcomes in patients remains uncertain. Compounding this, the rarity of the disease makes it difficult to assemble patient cohorts of sufficient size for meaningful clinical trials. At the molecular level, germline loss of SMARCB1 exons 4 and 5 has emerged as a particularly penetrant predisposing event, with affected individuals presenting at an earlier age than those harboring other mutation types. The porcine SMARCB1 gene offers a compelling basis for translational modeling as its protein product is identical to the human ortholog at every amino acid position across isoforms, a degree of conservation that exceeds what is seen in the mouse. **Methods:** Thus, we hypothesized that germline deletion of exons 4 and 5 would predispose heterozygote swine to ATRT development. In this manuscript, we describe the creation of an ATRT porcine model through a CRISPR/Cas9 mediated gene-editing approach. **Results:** 15 piglets were produced, two of which had confirmed *SMARCB1* targeted excisions. However, none developed tumors. To induce further tumorigenicity, one pig with confirmed exons 4 and 5 excision was crossed with a pig with *TP53* exon 2 truncation. In total, 11 piglets were born, of which one contained the original excision without a *TP53* mutation. This piglet developed a spinal mass at the T1 level. **Conclusion:** To our knowledge, this is the first ATRT porcine model ever developed and provides proof-of-concept feasibility for large animal modeling of SMARCB1-deficient rhabdoid tumors. These findings support the continued development of porcine RTPS-1 models toward preclinical application.

## Simple Summary

Rhabdoid tumor predisposition syndrome type 1 (RTPS-1) is a rare cancer predisposition syndrome that causes rhabdoid tumors, which are aggressive malignancies, predominantly in the central nervous system and kidneys. Progress to date for treatment of tumors arising from this syndrome has been limited in part due to the lack of preclinical models that faithfully recapitulate the disease. The aim of this study was to develop a large animal model of RTPS-1 through a CRISPR/Cas9 mediated gene-editing approach to achieve germline deletion of exons 4 and 5. We found that this approach induced tumorigenesis in a manner faithful to the human condition. Therefore, this model can be used to study tumor formation and other non-tumor phenotypes that will enhance understanding of the role that different *SMARCB1* genetic mutations play in humans.

## 1. Introduction

Atypical teratoid rhabdoid tumor (ATRT) is the most common malignant CNS tumor of children below 6 months of age. Although these aggressive tumors can sporadically occur, it can also be part of a rhabdoid tumor predisposition syndrome (RTPS), where rhabdoid tumors can occur in any anatomic location. There are two recognized entities; RTPS Type 1 (RTPS-1) is defined by *SMARCB1* mutation, whereas RTPS Type 2 (RTPS-2) is defined by *SMARCA4* mutations [1]. Patients with RTPS will usually present before 12 months of age with aggressive rhabdoid tumors that are extremely difficult to treat [2]. Due to the rarity of this condition making clinical trial recruitment challenging, there has been interest in developing preclinical models that can fully recapitulate the disease seen in patients.

One syngeneic mouse model employed *Rosa26Cre^ERT2^;Smarcb1^flox/flox^* to generate mice with temporal bi-allelic loss of *Smarcb1* that developed spontaneous rhabdoid tumors [3]. Another utilized co-inactivation of *p53* in a *GFAP-Cre;Snf5^flox/flox^;p53^flox/flox^* model to generate rhabdoid tumors [4]. Finally, our group developed a *P0-CreC;Smarcb1^flox/flox^* mouse model that also developed rhabdoid tumors early in development, confirming observations that ATRT development requires SMARCB1 inactivation in early embryogenesis between E6 and E10 and suggested a neural crest cell of origin [5]. While these models have improved our understanding of ATRT tumorigenesis, preclinical studies in mice are often not predictive of drug efficacy in humans, thus making translational studies difficult.

In contrast to mice, pigs (*Sus scrofa*) are similar to humans, including the central nervous system, which could be an ideal translational model [6]. With the completion of the porcine genome sequence, advances in precision gene targeting, and somatic cell nuclear transfer techniques, generating precise genetically modified porcine models is now feasible [7,8,9]. This opens up an array of new opportunities to study disease and develop novel and effective therapies, especially in rare conditions such as ATRT.

In humans, exons 5 and 9 in the *SMARCB1* gene appear to be hotspots for CNS tumors [10]. Furthermore, prior in-depth studies of human *SMARCB1* mutations demonstrated that exons 4 and 5 deletion predisposes patients to ATRT at a younger age; exons 4 and 5 deletion leads to a 5 kilobase excision, causing frameshift mutations of the canonical transcripts. As the genomic and protein structure of SMARCB1 is conserved between humans and pigs with 100% amino acid identity between the pig and human SMARCB1 isoforms, in pigs, exon 4 is differentially spliced and removing exon 5 results in a frameshift mutation [11].

In this study, we describe a RTPS-1 porcine model created by embryo microinjection of CRISPR/Cas9. The RTPS-1 porcine model exhibits spontaneous loss of heterozygosity (LOH), which is a critical step in rhabdoid tumorigenesis. We demonstrate that the RTPS-1 porcine model provides a unique opportunity to study the complex biology of ATRT and with further development and scalability may support preclinical evaluation of ATRT-targeted therapies as well as development of imaging methods and diagnostic biomarkers.

## 2. Methods

### 2.1. CRISPR Design, Synthesis, Validation

Target sites within the genes of interest were selected using CRISPOR [12]. The selection of guide RNAs targeting *SMARCB1* exons 4 and 5 was based on prior in-depth studies of human *SMARCB1* mutations demonstrating that germline deletion of exons 4 and 5 predisposes patients to ATRT at a younger age, producing an approximately 5 kilobase excision that introduces a frameshift in the canonical transcript [11]. Because porcine exon 4 is differentially spliced and removal of exon 5 results in a frameshift mutation analogous to that observed in humans [11], targeting this region was chosen to most faithfully recapitulate the most penetrant human RTPS-1 lesion. As a complementary, backup strategy, in the case that excisions were not efficient, a second strategy targeted exon 4 itself to generate frame-shifting indels that would be expected to similarly abrogate *SMARCB1* function [12,13,14,15]. Target sequences are available in Table S2. 

All gRNAs were synthesized through in vitro transcription and validated as previously described. Briefly, templates were purified (NucleoSpin Gel & PCR Cleanup, Macherey-Nagel), transcribed using the MEGAshortscript T7 kit (ThermoFisher Scientific, Waltham, MA, USA), and cleaned up with the MEGAclear kit (ThermoFisher Scientific, Waltham, MA, USA) followed by ammonium acetate precipitation. gRNA abundance was confirmed by Qubit fluorometric quantification (ThermoFisher Scientific, Waltham, MA, USA) [12,13,14,15]. 

### 2.2. Embryo Microinjection of CRISPR/Cas9

Embryos receiving different injection mixtures were unable to be maintained separately, and could only be differentiated later upon identification of the pattern of genome editing. Microinjections were performed by the Animal Models Core within the UW–Madison Biotechnology Center as previously described [15,16]. 

### 2.3. Estrus Synchronization, Superovulation, Artificial Insemination, and Embryo Retrieval from Donor Pigs

Estrus synchronization, superovulation, artificial insemination, and embryo retrieval were conducted as previously described. In brief, estrus was induced by intramuscular P.G. 600^®^ on Day-4, with standing heat assessed daily from Day-1. Follicular synchronization was achieved by prostaglandin F2α on Day 13, followed by superovulation 16 h later. Embryos were retrieved on Day 20 and cultured in porcine zygote medium (PZM3-MUI) prior to microinjection [15,16]. 

### 2.4. Estrus Synchronization, Embryo Transfer, Pregnancy, and Parturition in Surrogate Pigs

In surrogate pigs, estrus synchronization, embryo transfer, pregnancy, and parturition were conducted as previously described. In brief, surrogates synchronized to display estrus 18–24 h after donors received up to 150 presumptive zygotes per oviduct via laparotomy. Pregnancy was confirmed and tracked by transabdominal ultrasound, with farrowing expected at 115–117 days post-estrus [14,15,16,17].

### 2.5. Genotyping

Genotyping was conducted from tail biopsies of newborn piglets as previously described. Genomic DNA was extracted from tail biopsies by proteinase K digestion and isopropanol precipitation. Excision breakpoints were confirmed by Sanger sequencing, and indels and SNPs were quantified by targeted amplicon sequencing (TAm-Seq) on an Illumina MiSeq, analyzed with CRISPResso2 [15,18,19].

### 2.6. Copy Number Variant Analysis

Copy number variation (CNV) analysis was performed as previously described. Briefly, BamHI-digested genomic DNA was analyzed by droplet digital PCR (QX200 system, Bio-Rad) with primer and probe concentrations of 900 nM and 250 nM, respectively, using RPP30 as the reference gene for copy number normalization [15]. 

### 2.7. Immunohistochemistry and Tissue Staining

Hematoxylin and eosin staining was performed on 3.5 µm-thick sections prepared from paraffin blocks of formalin-fixed tissues. Immunohistochemistry was performed on the representative tumors. A standard protocol was used with primary antibodies incubated 1 hour at room temperature: SMARCB1 (1/200, BD Transduction Laboratories, 612110), ki-67 (1/2500, BD Transduction Laboratories, 556003), vimentin (1/200, Cell Signaling Technology, #5741), pan-Cytokeratin AE1/AE3 (1/300, Dako, M3515), neurofilament triplet proteins (1/500, Enzo Life Sciences, BML-NA1223), Sox10 (1/500, Cell Marque, 383R-14), S100 (1/5000, Dako, Z0311) and GFAP (1/3000, Dako, Z0334). Biotinylated secondary anti-rabbit or anti-mouse (Vector Laboratories, Burlingame, CA) IgG antibodies were incubated 30 min at room temperature. Immunoreactivity was quantified as the percentage of positively stained cells in four 40× tumor images (Positive cell detection module, QuPath version 0.6.0) or assessed based on the intensity of staining (absence, moderate or strong).

### 2.8. Statistical Analysis

Logistic regressions were performed in JMP (JMP Pro 15.0.0, SAS Institute Inc., Cary, NC, USA) using the presence or absence of germline transmission in progeny as the dependent categorical response and allelic abundance of the edited allele (pixel density or Illumina read representation) as the independent continuous regressor. Formal inferential statistics were not conducted due to small animal numbers.

## 3. Results

### Generation of a RTPS-1 Porcine Model Through CRISPR/Cas9 Editing

**Figure 1:**
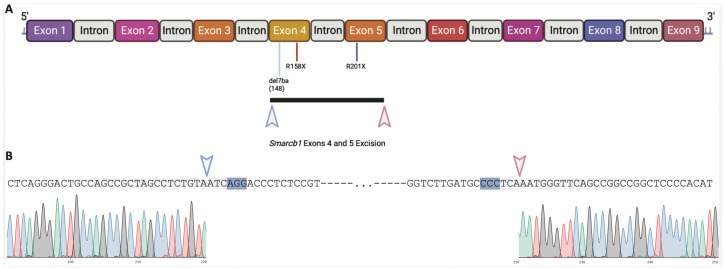
SMARCB1 excision. (**A**) SMARCB1 gene map and location of genetic alteration in the novel swine model. Bars note the mutations found in a human RTPS-1 cohort in exons 4 and 5. Arrows denote 5’ (blue) and 3’ (red) target sequences used to generate the exons 4 and 5 excision. (**B**) The sequence of the intronic regions flanking the excised exons 4 and 5 are shown, while Cas9 target sites (light blue) and PAMs (dark blue) are marked. Arrowheads indicated predicted target sites. Representative Sanger trace validates the excision.

**Figure 2:**
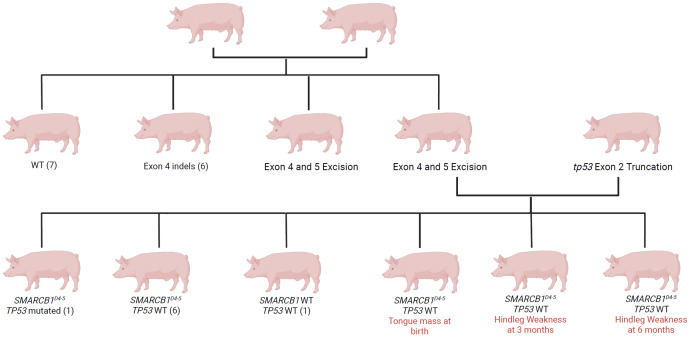
**Genogram of development of *SMARCB1* pig.** After embryo microinjection of CRISPR/Cas9 reagents into a surrogate pig, the first generation had 6 pigs with exon 4 indels, and 2 pigs with confirmed exons 4 and 5 excised. None developed tumors in the first 6 months. One pig with the confirmed exons 4 and 5 excision was crossed with a pig with confirmed p53 exon 2 truncation. In the second generation, 9 out of 11 pigs had a confirmed *SMARCB1* variant in exons 4 and 5. 1 pig had confirmed exons 4 and 5 excised and did not have a *TP53* mutation and developed a SMARCB1-negative spinal tumor identified due to hindleg weakness observed at 3 months. One pig had a tongue mass at birth that was not SMARCB1-negative. Finally, the other pig exhibited hindleg weakness at 6 months, but no mass was identified at necropsy. Other pigs did not develop any tumors by 6 months of age.

To generate a porcine model of RTPS-1, we set out to generate pig models with disrupted SMARCB1. To maximize the probability of recovering a preclinically relevant SMARCB1 alleles, complementary, parallel designs were implemented. The first was excision of exons 4 and 5, leading to a 5 kilobase excision, causing a frameshift mutation of the canonical transcript (Figure 1; Table S2). Other pigs were edited by inducing frameshifting indels into exon 4 (Table S2), which we predicted would be more efficient and cause a similar effect on SMARCB1 function. A total of 15 *F0* piglets were produced, which were then sequence validated (Table S1). Initial PCR and sequencing revealed 2 animals carried confirmed excisions of exons 4 and 5, while 6 animals carried indels introduced in exon 4. No piglets developed tumors after 6 months (Figure 2, Table S1).

To induce tumorigenicity, one *F0* pig with confirmed exon 4 and 5 excision was then bred with a pig with a confirmed *TP53* exon 2 truncation created in a previous study [15]. Out of 11 *F1* piglets, 1 carried the original excision. This unexpectedly low germline transmission rate led us to suspect this F0 pig carried cryptic mutations that evaded PCR and sequencing [15,20]. To investigate this possibility, we performed copy number variant assays querying both the 5’ and 3’ excision target sites (Table S2). The assay design would fail to detect a *SMARCB1* allele (“a copy”) if (a) the exon 4 and 5 excision was present or (b) some otherwise unidentified mutation modified the target sites and disrupted the queried region. CNV analysis revealed a patterned loss of copies that indicated a significant portion of the F0 pig’s germline carried at least two distinct SMARCB1 variants undetectable by our PCR assay: one variant disrupting both queried regions and one disrupting only the 3’ queried region. In total, 10/13 of the pig’s progeny carried an edited *SMARCB1* alelle.

**Figure 3:**
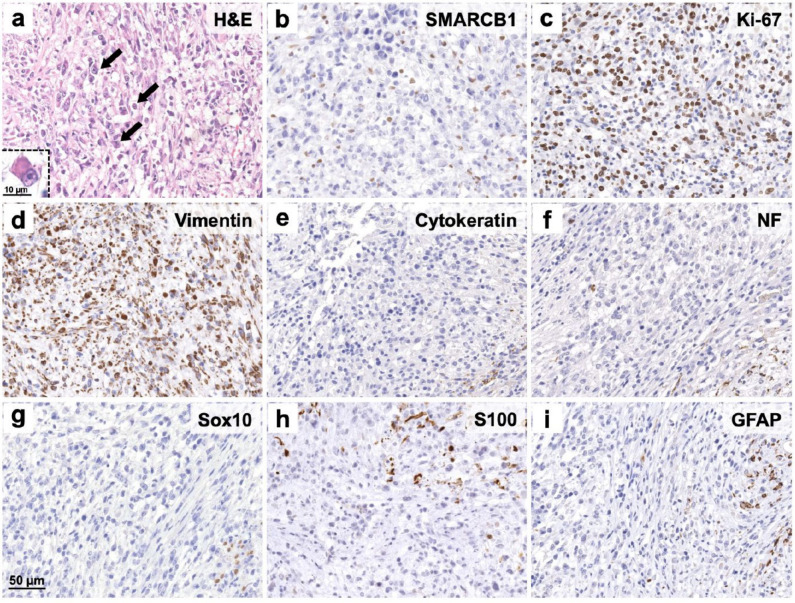
**Spinal tumor of the RTPS-1 pig displays typical histological and immunohistochemical staining features of human RTs.** (**a**) Tumor presenting with mesenchymal and vacuolar cytoplasmic degeneration pattern with classic rhabdoid cells with eccentrically placed nuclei and eosinophilic cytoplasmic inclusion (arrows and inset). (**b**) SMARCB1 staining shows loss of nuclear expression in tumor cells with retained expression in infiltrating inflammatory cells and intratumoral vasculature. (**c**) Ki-67 staining demonstrates high proliferative index. (**d**) Diffuse strong expression of vimentin in the tumor tissue. (**e**–**i**) No staining of cytokeratin (**e**), neurofilament triplet proteins (**f**), Sox10 (**g**), S100 (**h**), GFAP (**i**) noted in tumor cells but sparse positive cells are noted in entrapped residual normal cells.

The piglet with the exon 4 and 5 excision but without the *TP53* exon 2 truncation was identified at 2.5 months of age in the context of progressive hindleg weakness. On gross examination, the lesion appeared as a poorly demarcated, infiltrative soft tissue mass associated with the spinal canal. All histological markers are compatible with the diagnosis of rhabdoid tumor, including the presence of typical rhabdoid cells, absence of SMARCB1 staining in tumor cells, high Ki-67 staining (29.5 ± 5.8% of total cells), strong vimentin expression, and absence of other histological mimics such as Sox10, S100, and GFAP (Figure 3). One piglet with a *SMARCB1* variant developed facial abnormalities and a tongue mass that did not stain negative for SMARCB1. Finally, one piglet with a *SMARCB1* variant developed hindleg weakness; however, a mass was not identified. Piglets with phenotypic findings did not have the *TP53* mutation present.

## 4. Discussion

In this manuscript, we describe the first porcine model of SMARCB1-deficient RT with histological features consistent with ATRT. The tumor was negative for SMARCB1, suggesting spontaneous loss of heterozygosity (LOH), which is a critical step in disease progression. The tumor itself was negative for SMARCB1 by IHC, with retained expression in adjacent non-tumor cells serving as internal control, indicating potential somatic loss of the remaining wild-type SMARCB1 allele in the tumor. To our knowledge, a porcine model has not been developed for ATRT. Utilizing a CRISPR/Cas9 approach to delete exons 4 and 5 in keeping with the genetic aberrations and functional consequences found in human ATRT, we demonstrate a proof-of-concept large animal model that may be applicable for preclinical application and treatment evaluation for this rare tumor.

The tumor found in this CRISPR/Cas9-edited pigs displayed histological features like human ATRT, including aggressive growth and the presence of rhabdoid cells and absence of other principal histological mimics [21,22,23,24]. The anatomical locations have been described in humans in the past, including the spine and heart [22,23,24,25]. Furthermore, the affected pig with a spinal mass demonstrated neurologic signs and symptoms, including hindleg weakness. The pig was 2.5 months old when it started exhibiting symptoms, which is the equivalent to 12 months old in human years, which is consistent with the onset of ATRT in very young children and infants [24].

ATRTs are now categorized into three molecular subgroups: ATRT-SHH, ATRT-MYC, and ATRT-TYR. In particular, the ATRT-MYC subgroup is noted by broad deletions affecting Chromosome 22q11.2 and have been reported to occur in the lateral cerebellum, spine, and the head and neck region [25]. Unfortunately, due to the lack of tissue sample, we were unable to classify the tumor in one of the three subgroups, but given the later age of the piglet, tumor location, and broad gene deletion, the tumor could be closely related to the ATRT-MYC subgroup. Further studies will characterize these tumors through comprehensive molecular characterization, including DNA methylation array profiling, RNA-seq, and assessment of characteristic tumor signatures. 

It is notable that despite confirmed target excision in the first piglets in our original cohort, none of them developed tumors after 6 months, pointing to mosaicism, a known phenomenon in animal disease modeling and in the human condition [26]. We induced tumorigenesis by crossing a pig with the confirmed excision with another pig with a *TP53* mutation, which has been previously done in mouse modeling of ATRT by Ng, et al. to allow for permissive oncogenic background rather than to influence CRISPR editing of SMARCB1 [4]. Despite this crossing, piglets with phenotypic findings did not have the *TP53* mutation present; this may be attributable to the uncharacterizability of the *SMARCB1* allele in this pig. Nonetheless, our findings indicate that mosaicism and variant allele frequency play an instrumental role in ATRT tumorigenesis [26]. In this proof-of-concept model, the tumor-bearing pig may have carried a greater effective burden of SMARCB1 disruption than its conventional genotype suggested, which would lower the requirement of *TP53* co-mutation to initiate tumorigenesis.

Furthermore, the spinal cord arises from neural crest-derived and neuroepithelial lineages that are independently established as susceptible to SMARCB1 loss in early embryogenesis [5]. The location and timing of the observed tumor at the T1 spinal level at 2.5 months of age, developmentally equivalent to approximately 12 months of human age, is consistent with this cell-of-origin model and with the typical age of human ATRT.

In the future, somatic cell nuclear transfer (SCNT) represents another method by which porcine models could be generated. Although the efficiency is low, SCNT allows for more reliable complex genetic alterations and edits in comparison to embryo microinjections [27,28]. Moreover, using SCNT can reduce challenges like mosaicism and provide the ability to screen nuclei and enrich for the intended biallelic genotype prior to embryo generation [29,30]. However, the entry and cost barrier is high, and this must be considered in these large animal models.

## 5. Conclusions

In conclusion, the genetic, anatomic, and physiologic similarities between pigs and humans provide an ideal model for studying the biology of ATRT. Although the editing efficiency of the precise excision was a limitation of this first-generation model, the per-animal value of a confirmed RTPS-1 pig is high given the translational utility of a human-scale model. This proof-of-concept model suggests the potential to characterize tumor formation and possibly other non-tumor phenotypes that may help decipher the role of different *SMARCB1* genetic mutations found in humans. Through improvements in germline transmission efficiency and replication across additional animals, a scaled porcine RTPS-1 platform could support imaging studies, longitudinal biomarker sampling, and preclinical therapy evaluation. Future studies will focus on addressing the scalability and penetrance limitations of this first-generation model.

## Data Availability

The original contributions presented in this study are included in the article/Supplementary Material. Further inquiries can be directed to the corresponding author(s).

## Supplementary Materials

The following supporting information can be downloaded at https://www.mdpi.com/article/10.3390/cancers18121879/s1, Table S1: SMARCB1 Project-Subject Database, Table S2: Target Sequences.
