# Supplementary material for: Development of a Genetically Engineered Porcine Model of Rhabdoid Tumor Predisposition Syndrome Type 1 (RTPS-1)
Source: Cancers (Basel). 2026 Jun 9;18(12):1879. doi: 10.3390/cancers18121879 (PMC13297256; doi:10.3390/cancers18121879)
Supplement: Supplementary file 1 [file cancers-18-01879-s001.zip › cancers-4294267-table s2-Target Sequences.pdf]

Table S2

Target sequences		
Name	Target sequence (PAM)	Predicted cut site
target.5'.7	AGCCGCTAGCCTCTGTAATCAGG	Sscrofa11.1:14:49880366
target.ex4.1	CTGTTGATGGTCGTGGAGCAGGG	Sscrofa11.1:14:49880883
target.ex4.2	GCGGTTCCTGTTGATGGTCGTGG	Sscrofa11.1:14:49880889
target.3'.3	GGCCGGCTGAACCCATTTGAGGG	Sscrofa11.1:14:49883853
Primers		
Oligo name	Oligo sequence	IDT modifications
SMARCB1.ex4.F.1.6N.ILTS.1 (1802)	acactctttccctacacgacgctcttcc gatct NNNNNN TGTTGCCTCTGAGCCAGTGTG	TAm-seq
SMARCB1.ex4.R.1.ILTS.1 (1803)	gtgactggagttcagacgtgtgctcttc cgatct GCTGAGGGAGCTGGCTTCTG	TAm-seq
SMARCB1.5'.F.1.6N.ILTS.1 (1779)	acactctttccctacacgacgctcttcc gatct NNNNNN TTTGGAGCCCTGTGTGGGATG	TAm-seq and Sanger
SMARCB1.5'.R.1.ILTS.1 (1780)	gtgactggagttcagacgtgtgctcttc cgatct TGGCTCCTTGGAAGCACTGTC	TAm-seq
SMARCB1.3'.F.1.6N.ILTS.1 (1781)	acactctttccctacacgacgctcttcc gatct NNNNNN TTTGGAAGGTGTGGACTCTGG	TAm-seq
SMARCB1.3'.R.1.ILTS.1 (1782)	gtgactggagttcagacgtgtgctcttc cgatct GGGTGTCTGATCTCGGCTTTC	TAm-seq and Sanger
**CNV assays**		
Oligo name	Oligo sequence	IDT modifications
5'.probe.CNV.1 (2312)	AGGGACTGCCAGCCGCTAGCCTC	/56-FAM/AG GGA CTG C/ZEN/C AGC CGC TAG CCT C/3IABkFQ/
5'.F.CNV.1(2313)	CCGTGCGTCTAAAGTAATGC	unmodified
5'.R.CNV.1 (2314)	TAACAAGTCATGGGCTTGGC	unmodified

3'.probe.CNV.1 (2339)	CGGCCGGCTCCCCACATGTCC	/56-FAM/CG GCC GGC T/ZEN/C CCC ACA TGT CC/3IABkFQ/
3'.CNV.F.1 (2340)	CAGAGTGTGACGAGGGTCT	unmodified
3'.CNV.R.1 (2341)	GACCAAGGAAGTGACAGAGG	unmodified
